# Review on the Formulation, Existing Problems, and Practical Effects of Fitness Exercise Prescriptions for People With Intellectual Disabilities

**DOI:** 10.3389/fpubh.2022.936830

**Published:** 2022-07-22

**Authors:** Zengyin Yan, Pingping Yan, Chunli Qin, Jiong Luo

**Affiliations:** ^1^School of Physical Education, Chongqing University of Posts and Telecommunications, Chongqing, China; ^2^The College of Exercise Medicine, Chongqing Medical University, Chongqing, China; ^3^Chongqing Institute of Sport Science, Chongqing, China; ^4^College of Physical Education, Southwest University, Chongqing, China

**Keywords:** intellectual disabilities, exercise prescription, cardiopulmonary fitness, muscle fitness, body composition

## Abstract

Compared with normal people, patients with intellectual disability have poor cardiopulmonary and muscle fitness levels, and their daily physical activity generally cannot reach the “guideline-recommended amount,” which increases the risk of obesity and cardiovascular disease in this group. From the perspective of six elements of exercise prescription (frequency, intensity, time, form of exercise, amount of exercise, and progressive rate), this paper systematically reviews the current situation of the formulation and implementation of exercise prescription for patients with intellectual disabilities. The results show that the design idea of aerobic fitness exercise prescription for patients with intellectual impairment follows the six-element ^5^paradigm, but the insufficient recommended amount of each element is a common problem. In the design of muscle fitness exercise prescription, due to the differences of different exercise forms, the description of the six elements is very inconsistent. Although most prescription execution effects show that it is beneficial to improve cardiopulmonary and muscle fitness, there is a great debate on whether it is beneficial to improve body composition. People with intellectual disabilities are highly heterogeneous groups. In the initial stage of exercise intervention, the elements of exercise prescription need to be adjusted individually to obtain sustainable positive benefits.

## Introduction

Compared with normal people, people with intellectual disabilities have poor cardiopulmonary fitness and muscle strength (1, 2), a serious lack of physical activity, and a significant increase in static sedentary time (3, 4). These factors can easily lead to metabolic syndromes, such as overweight/obesity, hyperlipidemia, and^12^ diabetes (5–7). The outline of the “healthy China 2030” plan points out to carry out national fitness activities and promote sports activities for key groups. At present, the number of intellectual disabilities in China is huge. There is an urgent need to change their sedentary lifestyle, reduce the incidence of cardiovascular disease, reduce psychological stress, and improve depression through appropriate health promotion plans (8, 9). Exercise is one of the main means to effectively reduce the incidence of the above diseases, and an exercise prescription is an individualized exercise plan. Its purpose is to enable individuals to obtain better cardiopulmonary and muscle fitness. It needs to be optimized and adjusted according to individual health level, physical activity ability, and physical fitness status. Therefore, exercise prescription is considered to apply to special groups with chronic diseases and physical and mental disorders (10). According to the norms in the guidelines on exercise testing and exercise prescription issued by the American Sports Medical Association (ACSM), the exercise frequency, exercise intensity, exercise time, exercise form, exercise volume, and the progressive rate are regarded as the golden criteria of the fitness industry (frequency, intensity, time, and type [FITT]-VP). At the same time, they are also recognized by many scholars at home and abroad and serve as the basic criteria for people with intellectual disabilities to formulate exercise prescriptions (11, 12). Therefore, this paper has taken people with intellectual disabilities as an example, reviewed the current situation of their physical fitness, systematically reviewed the literature related to the intervention of exercise prescription to promote the healthy physical fitness of people with intellectual disabilities, found out the advantages and disadvantages, and then provided a theoretical and practical reference for improving the prescription design.

## Data and Methods

### Data Sources

Using PubMed, MEDLINE, web of science, Cochrane, China HowNet, dimensional spectrum, and other databases, search for research papers was published in relevant journals at home and abroad from January 2005 to December 2021 by using keywords, such as intelligent disorder, exercise prescription, healthy physical fitness, aerobic, muscle endurance, muscle strength, flexibility, physical activity, exercise, training, progressive rate, and so on.

### Finalist Criteria

1) The subjects were patients with a group of clinical syndromes, namely, memory, cognition (generalization, calculation, judgment, etc.), language, visuospatial function, and personality, were impaired; 2) the experimental group had a strict exercise prescription design and included the control group at the same time; 3) the prescription included aerobic training, resistance training, flexibility training, and partial resistance training; and 4) the prescription design was following the standards of American College of Sports Medicine (ACSM). The evaluation indexes mainly included form, function, and physical quality.

### Literature Exclusion Criteria

1) Documents whose language was not English or Chinese were excluded; 2) not excluded by randomized controlled trial; 3) repeated and non-experimental studies were excluded; 4) nonmotor intervention exclusion; and 5) to explore the effectiveness of non-healthy physical fitness and nonintelligent disorders.

### Data Intake Quality Assessment

1) The shortlisted literature was read in three stages. In the first stage, a researcher searched the database and browsed the title and abstract, and preliminarily selected the documents found. In the second stage, another researcher arranged the literature to eliminate duplicate literature. In the third stage, two researchers read the full text together to determine whether the literature met the inclusion criteria. If there was any literature that had not reached a consensus, it was decided after discussion.
2) Literature quality and empirical level. The Physiotherapy Evidence Database (PEDro)-scale was used to check each document and evaluate its research quality. The higher the score, the better the research quality of this document. Each document was scored independently by two researchers. If there were different scoring items, a consensus was reached after discussion. Due to the characteristics of the included papers, the therapists were required to provide treatment intervention in the research process. The maximum total scores of the items that could not be single-blind to the therapists maybe 9 points. Therefore, it was determined that those whose PEDro-scale score was > or equal to 5 points were high-quality papers and those whose score was < or equal to 4 points were low-quality papers.

**Figure 1:**
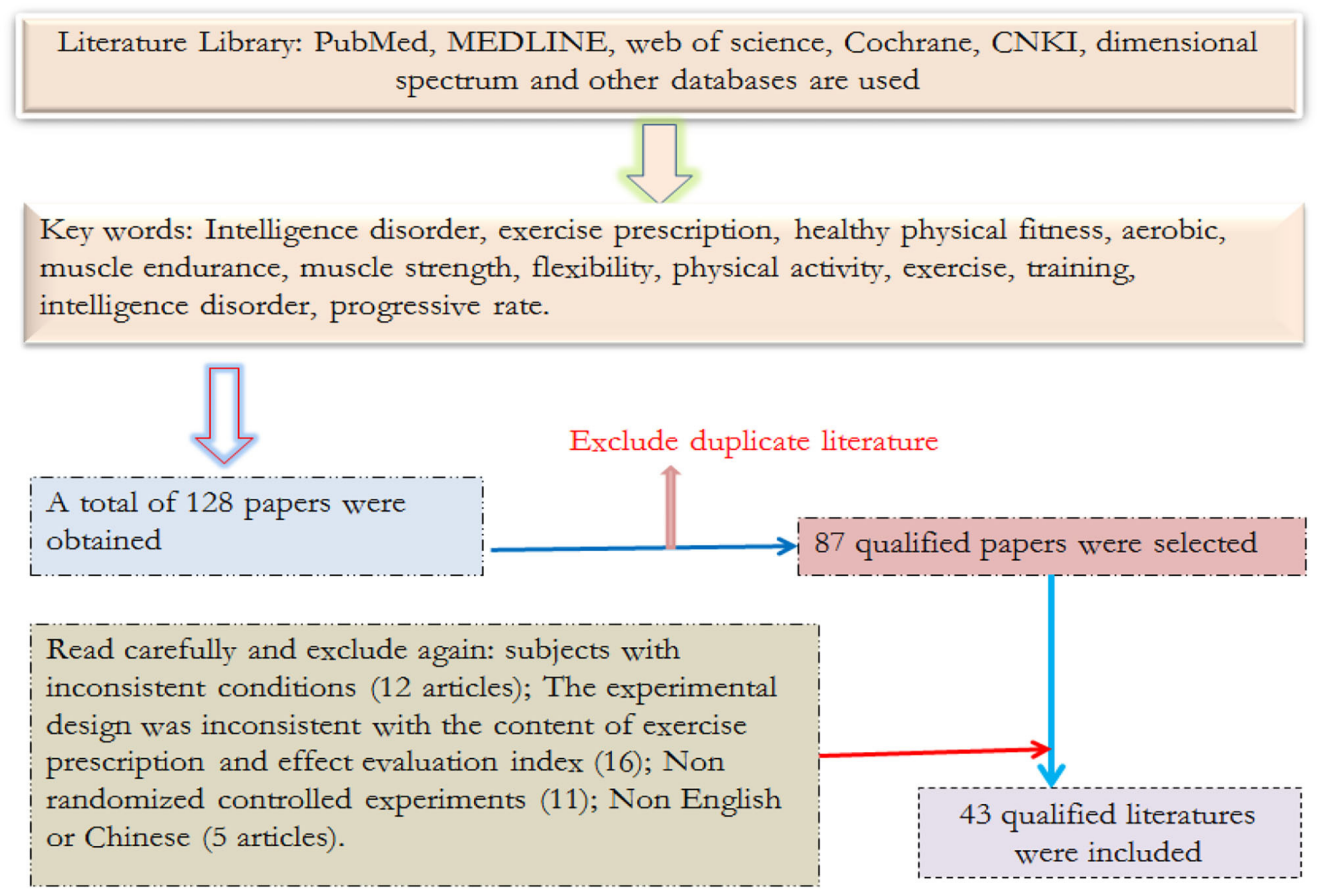
Schematic diagram of literature screening.

According to the keyword search strategy, a total of 128 relevant literature were found, 41 duplicate literature were excluded, and the remaining 87 kinds of literature were included. After browsing the title and abstract, 44 inconsistent with the literature selection criteria were excluded, 12 kinds of literature did not meet the conditions of subjects, 16 experiments were designed to be inconsistent with the contents of sports treatment and effect evaluation indexes, 11 were non-randomized controlled trials, and 5 kinds of literature were not in English or Chinese). Finally, 43 pieces of research literature were included for comprehensive analysis. The process of literature search and inclusion in this study is shown in Figure 1.

## Results and Discussion

### Physical Fitness Status of People With Intellectual Disabilities and Suggestions on ACSM Prescription

#### Physical Fitness Performance Status of People With Intellectual Disabilities

The Study Found That the Cardiopulmonary Fitness of Children and Adolescents With Intellectual Disabilities Is Significantly Lower Than That of Non-intellectual Disabilities. They Have Lower Peak Oxygen Intake (VO_2Peak_) and Lower Peak Heart Rate (HR_peak_), Which Will Gradually Decrease With age. Among Them, Women With Intellectual Disabilities Have Lower Cardiopulmonary Function Than men (13). Hartman et al. (14) Compared the Performance of Physical Fitness Between 8 and 12-Year-Old Children With General (*n* = 515) and Children With Intellectual Disabilities (*n* = 73), and Conducted a 4-Year Longitudinal Study on Children With Intellectual Disabilities, Which Found That General Children Were Significantly Better Than Children With Intellectual Disabilities in Running Speed, Aerobic Endurance, Explosive Power, Grip Strength, and Abdominal Muscle Endurance. Boonman et al. (15) Compared the Cardiopulmonary Function of 27 People With Intellectual Disabilities and 35 Adults With Normal Intelligence. The Results Showed That People With Intellectual Disabilities Had Lower HR_peak_ and VO_2peak_. The HR_peak_ of Intellectual Disabilities Is About 8–20% Lower Than That of Ordinary People. Up to 50% of Intellectual Disabilities Have Congenital Heart Disease, and Their Cardiopulmonary Fitness Level may be About 50% Lower Than That of the Same age With Normal Intelligence, Which Shows That the Cardiopulmonary Fitness of People With Intellectual Disabilities Is Lower Than That of Ordinary People (16, 17). Compared With Normal People, the Knee Flexion and Extension Torque, leg, and Back Muscle Strength of 8–18-Year-Old People With Intellectual Disabilities Are Significantly Weaker, and the Stability of the Knee Joint Is Also Worse Than That of Normal People. The Reason Is That the Isokinetic Muscle Strength and Ratio (H/Q) of the Front and Rear Thigh Muscles of People With Intellectual Disabilities Are Lower (18, 19). It can be Seen That the Muscle Fitness of People With Intellectual Disabilities Is Still Weaker Than That of Ordinary People. Maiano's Integrative Analysis Showed That (20) When Compared With Normal Peers, the Overweight or Obesity Rate of People With Intellectual Disabilities Was Nearly two Times, While the Obesity Rates of Children and Adolescents With Intellectual Disabilities Was 13 and 15%, Respectively. Carter and Swan (21) Found That the Prevalence Rates of Obesity in Primary School, Junior Middle School, and Senior High School Were 9.2, 18.6, and 26.9%, Respectively; de Winter et al. (22) Found That the Proportions of Overweight and Obesity Among People With Intellectual Disabilities Aged 60 Were 38 and 26%, Respectively, While Only About 10% of the General Elderly.

#### ACSM Prescription Suggestions for People With Intellectual Disabilities

Table 1 shows that:

1) Maximum oxygen uptake is regarded as the most reliable index to evaluate cardiopulmonary function. The American Sports Medical Association suggests that people with low fitness should adopt an appropriate heart rate of 50–70%, and people with physical and intellectual disabilities should adopt a 40–80% exercise intensity. No matter which one is adopted, they should adopt a gradual approach to the indicators provided by the recommended policy: the exercise intensity should be 60–80% of the maximum heart rate, and about 8–20% of people with intellectual disabilities should underestimate the maximum heart rate, that is, the heart rate per min is 10–15 times lower than the expected standard. The average maximum heart rate of patients with Down syndrome may be <30–35 beats per min. Non-maximal exercise test is often used in practice tests to estimate the maximum oxygen uptake of intellectual disabilities, such as common step-up test, 20 m cardiopulmonary endurance run, and 1,600 m endurance run, which are highly related to the maximum oxygen uptake, so it can effectively evaluate the cardiopulmonary fitness at this stage.
2) Muscle strength and muscle endurance are regarded as two important indicators to evaluate muscle fitness. The improvement of muscle fitness generally follows the principle of progressive overload, that is, gradually adjusting the repetition times and load weight of specific muscle groups. There are three types of trainings, i.e., isotonic muscle strength, isometric muscle strength, and isokinetic muscle strength. Considering the low participation motivation and lack of understanding of attention and tasks of people with intellectual disabilities, weight-bearing training is not recommended in the basic stage of exercise prescription, because congenital muscle tension is poor, resulting in rapid muscle fatigue and easy withdrawal of cases from the plan. Down's congenital musculoskeletal abnormalities and poor joint stability can also lead to excessive weight-bearing. However, the improvement of muscle fitness has a significant effect on cardiopulmonary capacity (23), especially lower limb training and aerobic exercise (such as the flywheel, hiking, and running) are very important to cultivate muscle fitness.
3) Sitting forward flexion is the most common important index to evaluate flexibility. Flexibility refers to the movable range of the joint, and the tolerable range is the appropriate stretching intensity. After reaching the maximum length of the stretching muscle, you will feel weak pain, and it is appropriate to continue the stretching action. The flexibility of each joint has great particularity due to its structure and characteristics. For example, the shoulder joint can be used for flexible activities in three-dimensional space and can be used for large stretching actions. The elbow joint and ankle joint can only move on one plane. If the intellectual disabilities are accompanied by cerebral palsy, they need a joint protractor, and a doctor or physical therapist will issue a prescription, and parents, teachers, or professionals will assist in joint rehabilitation.
4) Body composition is an important index commonly used to evaluate body composition. Although the physical composition is not mentioned in the physical fitness assessment and exercises prescription provided by ACSM to the people with chronic diseases and physical disabilities, relevant studies have found that obesity has a high correlation with coronary heart disease, stroke, arteriosclerosis, and diabetes (24, 25), and the structured aerobic exercise and muscle fitness training prescription can effectively reduce the obesity, triglyceride, total cholesterol, and low-density lipoprotein of young people with intellectual disabilities. It also shows the necessity of formulating appropriate exercise prescription.
5) According to the gradual principle of exercise prescription, the general training is divided into a basic period (4–6 weeks), a progressive period (5–6 months), and a maintenance period. If the patient's physical fitness is poor, start the intervention with a shorter exercise time (10–15 min), gradually increase to 5 days/week, and continue the aerobic activity for 30 min/time. The basic period is to be familiar with skills, operating equipment, understanding safety matters, etc. After entering the progress period, it will increase the distance or shorten the time by the same distance. Maintenance period refers to the period when the range of progress slows down or stagnates after the gradual increase of intensity and exercise time. It can judge the effective exercise intensity, frequency, and time, focusing on maintaining the current progress results. Aerobic activities with low impact and low technical content (such as swimming, hiking, and flywheel) are mainly used. The combined training of 1–3 groups of major muscle groups was performed two times a week, and each group was repeated 8–12 times. The training effect will make significant progress in 10–12 weeks, and it will take 4–6 months for cardiopulmonary fitness.

**Table 1:** The American Sports Medical Association (ACSM) exercise prescription recommendations for people with intellectual disabilities.

**Type**	**Target**	**Strength / maximum load / extension**	**Frequency**	**Exercise time**	**Intervention cycle/ progression rate**
Aerobic exercise (mainly walking–gradual to intermittent running, swimming, power exercise bike)	Weight control; Improve cardiopulmonary fitness; Improve load capacity	40–80% maximum oxygen uptake;40–80% of the maximum heartbeat;3–7 days a week, 20–60 min each time	3–7 d/ week to maximize heat consumption;	30–60 min / day; 10~15 min Min intermittent exercise	The 4–6 month progressive rate depends on the situation of the case
Resistance exercise (equipment training is more suitable for free weight; heavy training equipment; equal length training; elastic rope; weight–bearing training)	Improve muscle strength and endurance of different muscle groups	60–70% of 1RM was initially used, and it was adjusted to 75–80% of 1RM after 1–2 weeks; 70–80% of the maximum load 2–3 times a week for three rounds, 8–12 times per round, with an interval of 1–2 min, rest to avoid injury.	2–3 days / week	2–3 groups of training were conducted for the main muscle groups; 12 times / 1 round (initial stage); Rest for 1–2 min / round	The progressive rate of 0–12 weeks depends on the situation of the case (such as gradually increasing the intensity; after 2 weeks, it is carried out with 80% of the maximum muscle strength; after 4 weeks, it is increased to 2–3 groups of training rounds)
Flexibility passive stretching (with human assistance); Active stretching	Improve the mobility of specific joints	Stretch to a slightly uncomfortable degree; 3–5 days/week, repeat 3–5 times, ≤ 30 s/time.	At least 2–3 days/week	Each action can be repeated 2–4 times; The action lasts for 10–30 s	10–12 weeks, none (unstable atlantoaxial joint of neck, avoid excessive extension of neck)

*HRR, heart rate reserve; VO_2_R, oxygen uptake reserve; RPE, rating of perceived exertion; 1RM, one repetition maximum*.

### Analysis of the Effect of Exercise Prescription Intervention in Fitness Practice of People With Intellectual Disabilities

According to ACSM recommendations, low-risk groups can take the maximum exercise test or participate in intense exercise and moderate-risk groups can take the non-maximum exercise test or appropriate exercise plan. If necessary, it is recommended to be supervised by doctors. However, cases assessed as high-risk groups cannot take exercise evaluation or exercise plan without the presence or consent of doctors. The formulation of exercise prescription is written in subjective condition, objective condition, evaluation and analysis results, and plan (SOAP) mode implementation. A perfect exercise prescription includes a physical fitness evaluation and an exercise plan. After evaluation, we can understand the existing ability. The evaluation focuses on cardiopulmonary fitness, muscle fitness, softness, and body composition. The basic elements of designing an exercise prescription include six elements: exercise frequency, exercise intensity, exercise time, exercise form, exercise amount, and progressive rate. According to its English expression, the prefix combination is FITT-VP.

#### Current Situation and Effect Analysis of Aerobic Exercise Prescription Intervention

Table 2 shows that

1) The prescription design of most experiments does not clearly explain whether to follow the recommended standard of ACSM prescription. The intensity (light and medium intensity) and intervention cycle (12–13 weeks) are the most, while the obstacle degree of the research objects is more than moderate and severe, and the exercise type is mainly walking (30). From the perspective of prescription effect, although many schemes do not fully meet the recommended conditions of exercise prescription, they still have achieved certain intervention effects but there is a lack of clear explanation for the interaction mechanism and influencing factors of intensity and intervention cycle.
2) The health benefits of aerobic exercise (such as walking and road running) are the same. The performance is that cardiopulmonary fitness and muscle endurance have been significantly improved, muscle strength has also been significantly improved, and the improvement of flexibility is related to the exercise mode. In the body composition, most studies found that body weight and BMI had a little obvious effect after intervention (26, 27, 29, 31); however, Boer and moss (28) used indoor bicycles and treadmills for intermittent and continuous aerobic exercise for patients with Down's disease. The results showed that both modes can effectively reduce weight. In addition, whether the subjects are Down's or non-Down's intellectual disabilities, intermittent sprint training and continuous aerobic training can improve the subjects' weight, percentage of body fat, and BMI after 12 weeks of exercise intervention (32, 33). Therefore, it is speculated that the exercise prescription, intermittent training, and medium-intensity continuous exercise intervention for patients with intellectual disability, if lasting for 12 weeks or more, should effectively improve cardiopulmonary fitness and body composition, but the exact mechanism is not clear.

**Table 2:** Effects of an exercise intervention on physical fitness and body composition of persons with intellectual disabilities.

**References**	**Object characteristics**	**Motion type**	**Exercise prescription items**		**Intervention effect**
			**Fit**	**Unfit**	
Wouters et al. (2)	Moderate and severe youth	Road running	For 16 weeks, the exercise intensity was a mixture of low, medium and high intensity, three times a week, 50 min each time (5/5).	/	Muscle strength, muscle endurance and cardiopulmonary fitness were significant, but BMI and flexibility were not significant.
Skelly et al. (26)	Moderate adult	15 m go	24 weeks, 5 times every 2 weeks, 35 min each time (3/5).	The recommended quantity of strength and frequency is not reached	Flexibility, leg strength and cardiopulmonary fitness have improved significantly; Body fat decreased, but BMI and waist hip ratio did not reach a significant level.
Boer et al. (24)	Mild young people	Aerobic apparatus	15 weeks, twice a week, 40 min each time. SIT(2/5), continuous aerobic training (2/5).	The frequency and cycle are not up to the recommended amount, and there is no strength description	Both SIT and continuous aerobic training are effective in improving cardiopulmonary fitness, waist circumference and fat percentage, but SIT is better in improving cardiopulmonary fitness and body composition
Yuhua and Yongji (27)	Moderate and severe youth	Ramp walking	For 12 weeks, twice a week for 30 min each time, the maximum heart rate needs to be more than 130 beats (3/5)	Progressive rate and frequency are not mentioned; The intervention cycle did not reach the recommended amount	It is effective in improving cardiopulmonary fitness and muscle endurance; It has no effect on BMI improvement.
Boer and Moss (28)	Down syndrome adults	Aerobic apparatus	12 weeks, 3 times a week, 30 min each time. Intermittent training (3/5), continuous aerobic training (4/5)	Low intensity intermittent; Both kinds of training did not reach the recommended amount of cycle	Interval training is more effective for body weight and BMI; Continuous aerobic training is more effective for functional test and leg muscle strength; Both groups were effective in improving cardiopulmonary fitness.
Zhongying et al. (29)	Severe adult	15 m go	For 12 weeks, exercise 5 times every 2 weeks, each time for 35 min (2/5)	The intensity, frequency and period did not reach the recommended amount	Flexibility (right ankle, left knee joint) and cardiopulmonary fitness are significantly effective; Flexibility, body composition, muscle strength, cardiopulmonary fitness have significant results

*BMI, body mass index: SIT, sprint interval training*.

In conclusion, when using an aerobic exercise prescription, even if the intensity is low and the exercise frequency is 2 times/week, if the continuous exercise with intervention time ≥12 weeks, it can significantly improve cardiopulmonary fitness, muscle strength, and muscle endurance. There is no consistent conclusion on body composition or BMI, which may be related to how to scientifically combine the six elements of FITT-VP in the design of exercise prescription. It is reflected in that most of the intensity and intervention cycles do not meet the recommended amount of ACSM (for example, 40–80% medium and high-intensity reserve heartbeat and reserve oxygen uptake or a 4-month intervention cycle). In addition, for people with intellectual disabilities, the initial standards of various elements of exercise prescription are suggested. However, due to the highly heterogeneous physical and mental characteristics of people with intellectual disabilities (such as movement development, physical fitness status, and participation motivation), in the initial stage of participating in exercise, exercise prescription may still need to be optimized and adjusted according to individual current situation ability.

#### Effect Analysis of Muscle Strength and Muscle Endurance Exercise Intervention

Table 3 shows that when foreign scholars train muscle strength and muscle endurance for people with intellectual disabilities, their prescription design mostly adopts different combination training methods, and the training frequency follows the guide recommendations, but most do not mention the maximum load (10). In terms of the effect of sports intervention, most scholars have affirmed that resistance or instrument training can improve muscle strength, muscle endurance, and muscle mass (34–36), but in terms of body composition, there is no reduction in body fat rate and significant improvement in BMI (40). In terms of training methods, we mainly carry out unarmed training for the required muscle groups (34) and the operation of upper and lower limbs (35), such as using pulley pull-down, sitting chest push, sitting rowing, and other actions for the upper limbs. For lower limbs, use sitting thigh push and pedal, sitting leg extension, sitting heel lifting, and other actions. At present, few domestic scholars choose to use equipment training as a way of sports intervention for people with intellectual disabilities, but adopt more conservative training methods, such as unarmed resistance training, sit-ups, or non-dangerous equipment, such as an elastic rope to replace weight training equipment, to avoid injuries caused by excessive use of muscles by people with intellectual disabilities (38, 39). Cai Hongqi et al. (39) conducted 12 weeks of stretching training for students with moderate and severe intellectual disabilities. The results showed that the flexibility, dynamic and static balance, and agility of the subjects were significantly improved, and the above sports results could be maintained after 4 weeks of training suspension; Wu Yujie (38) used the Total Body Resistance Exercise (TRX) suspension method to carry out 45-min suspension training on the core muscles of students with intellectual disabilities for 8 weeks, three times a week. The results show that TRX suspension training can significantly improve the core muscle fitness of students with intellectual disabilities—abdominal muscle strength and back muscle endurance.

**Table 3:** Effects of an exercise intervention on muscle strength, muscle endurance, and flexibility of people with intellectual disabilities.

**References**	**Object characteristics**	**Motion type**	**Exercise prescription items**		**Intervention effect**
			**Fit**	**Unfit**	
Gupta et al. (34)	Children and adolescents with Down syndrome	Progressive resistance training (sandbag)	6 weeks, 3 times/week, 10 repetitions in 2 groups, and increase resistance after reaching (2/5)	The maximum load, time and cycle do not reach the recommended amount	The strength of hip flexor, hip extensor, hip abductor, knee flexor, knee extensor and ankle plantar flexor were significantly increased compared with the control group
Shields and Taylor (35)	Down syndrome adolescents	Upper and lower limb instrument training	10 weeks, 2 times/week, 3 groups each time, 12 repetitions, increase resistance after reaching (5/5)	/	The lower limb muscle strength was significantly improved, while the upper limb muscle strength was not significant; The strength effect of upper and lower limbs is similar, which is medium effect.
Kao & Wang (36)	Mild to moderate youth	Unarmed, elastic band	13 weeks, 2 times/week,10 times per action, 2 cycles, a total of 40 min (3/5)	The recommended amount of time is not reached and the maximum load is unknown	It can improve the strength, movement speed and balance ability of lower limbs; The improvement of load–bearing capacity is not significant.
Mokhlesin et al. (37)	Special education class students	Core muscle training and bowling training	12 weeks, 3 times / week, 15 min each time (2 / 5)	The maximum load, time and progressive rate are not described	The influence of body composition is inconsistent. BMI did not achieve significant improvement; Body fat rate decreased and muscle mass increased.
Yujie (38)	Mild to moderate youth	TRX suspension training	8 weeks, 3 times/week, 50 min each time (2/5)	The recommended amount of intervention cycle is not reached, and the maximum load, progressive rate and time are unknown	Abdominal muscle strength and back muscle endurance were significantly improved
Hongqi et al. (39)	Moderate to severe adult	Stretching exercise	12 weeks, 3 times / week, 50 min / time (5 / 5)	/	Dynamic and static balance, agility and flexibility are significantly improved; The exercise effect still exists after 4 weeks of training suspension.

In short, at the initial stage of training, the use of equipment training is safer than unarmed resistance training, and the effect of equipment training is more obvious in terms of muscle endurance and muscle strength. Considering the low participation motivation and lack of understanding of attention and tasks of people with intellectual disabilities, weight-bearing training is not recommended in the initial stage of exercise prescription, because the congenital poor muscle tension makes the muscles tired quickly and is easy to cause the case to withdraw from the plan.

#### Effect Analysis of Combined Sports and Multi-Element Intervention

Table 4 shows that at present, the combined exercise intervention of foreign scholars for people with intellectual disabilities mainly includes the combination of different exercise forms, such as aerobic, muscle, and muscle endurance and flexibility, while the multi-element intervention is reflected in the multi-element combination, such as health education, nutrition education, community resources, and peer guidance (42, 44). It is a strategy often used in the field of sports promotion for people with physical and mental disabilities in recent years. For people with intellectual disabilities, the methods adopted by Chinese scholars are similar to those adopted by foreign scholars. They also use combined exercise or multi-element intervention to promote their health and physical fitness (43). Through a systematic review of these exercise prescription effects, they can be roughly divided into three categories: after exercise intervention, the cardiopulmonary and muscle fitness of people with intellectual impairment have made significant progress, the flexibility has not reached significant (41), and the BMI is inconsistent (41, 43). Bouzas et al. (10) systematically reviewed the effects of 44 exercise interventions on adults with mild and moderate intellectual disability and found that aerobic exercise, muscle strength, and muscle endurance exercise or combined exercise are the most common intervention methods. These exercises have positive effects on the cardiopulmonary and muscle fitness of people with intellectual disabilities. Half of the studies also discussed the impact of an exercise intervention on body composition, of which about 50% are effective. When conducting exercise intervention for flexibility items, such as muscle fitness exercise intervention, it is mostly combined with different physical fitness elements or specific motor skill training to plan exercise plans and courses and then explored the impact of the intervention on flexibility.

**Table 4:** Effects of combined exercise and other exercise interventions on physical fitness of people with intellectual disabilities.

**References**	**Object characteristics**	**Motion type**	**Exercise prescription items**		**Intervention effect**
			**Fit**	**Unfit**	
Barwick et al. (40)	Special Olympics athletes	Functional training group: muscle strength and muscle endurance (unarmed), aerobic and flexibility; Weight training group: muscle strength and muscle endurance (equipment), aerobic and flexibility	12 weeks, 2 times / week, 30 min / time (muscle), 20 min (aerobic), 10 min (flexibility). Aerobic (1 / 5), muscle (4 / 5), flexibility (2 / 5)	Aerobic: the frequency, time and intervention cycle did not reach the recommended amount; The strength and progressive rate are unknown; The muscle strength did not reach the recommended amount; Flexibility does not describe strength, time, and rate of progression.	The cardiopulmonary endurance before and after functional training group was significantly improved, and the maximum heart rate was significantly reduced; Compared with the weight training group, the duration of abdominal static muscle endurance was significantly prolonged.
Calders et al. (23)	Mild and moderate youth	Aerobic and resistance training machine, warm–up and relaxation exercise	20 weeks, twice a week, 70 min each time (including oxygen and muscle), with a gradual rate. Aerobic (4 / 5), muscle (5 / 5)	Aerobic: the frequency does not reach the recommended amount	Aerobic endurance and combined exercise have significant effects on the improvement of physiology and muscle strength; However, combined exercise is better than aerobic endurance in physiology and muscle fitness (upper and lower limbs, abdominal muscles and grip strength); Both in BMI, waist circumference did not reach significant.
Collins and Staples (41)	Mild to severe children	Structured physical education courses (gross movements and their associated motor skills training)	10 weeks, once a week, 90 min each time (1 / 5)	The intensity, frequency, intervention cycle and progressive rate are unknown	Aerobic capacity, muscle strength and muscle endurance have improved significantly, but the flexibility of left and right feet has not improved significantly
van Schijndel–Speet et al. (42)	Mild to severe adult	Physical activity courses (muscle strength and endurance, flexibility, sense of balance), health promotion program.	8 months, 3 times / week, extended for 45 min after 20 min. Aerobic (4 / 5), muscle and flexibility (3 / 5)	The aerobic strength does not reach the recommended amount; Muscle and flexibility do not specify strength and time	It has significant effects on muscle strength, blood pressure, serum cholesterol and cognitive function; Body weight, waist circumference, walking speed and blood glucose were not significantly improved.
Meihua et al. (43)	Middle and severe primary school special education class students	Physical fitness exercise course	12 weeks, 3 times a week, 40 min each time. Aerobic (2 / 5), muscle and flexibility (3 / 5)	Aerobic: the time and intervention cycle did not reach the recommended amount; The strength and gradual rate are unknown; Muscle and flexibility do not specify strength and time.	Cardiopulmonary endurance, flexibility, abdominal muscle strength and muscle endurance were effective, but BMI was not significantly improved

In short, from the six elements of FITT-VP, the prescription type, frequency, time, and intervention cycle are mostly unable to describe the intensity of muscle strength and muscle endurance exercise intervention, such as maximum load, time, and gradual rate. The reason may be that when compared with the resistance training, the maximum load can be known by using the equipment, but when the intervention content is not easy to standardize and measure, for example, the resistance parameters of the elastic rope are unknown due to the different manufacturers of the elastic rope, so the maximum load cannot be described.

## Conclusions and Suggestions

### Conclusions

Compared with normal people, people with intellectual disabilities are faced with poor physical fitness. Although the six elements of FITT-VP cannot be used to plan exercise prescriptions in all six aspects, the cardiopulmonary fitness, muscle fitness, and flexibility of people with intellectual disabilities can be significantly improved. Only the effect of changing body composition is inconsistent. Whether using a single exercise mode or using combined exercise or multiple element interventions, when planning exercise intervention, it is still necessary to make optimization adjustments according to the action development and physical fitness status of the intellectual disabilities and then gradually reach the recommended standards for each element of the exercise prescription of the intellectual disabilities.

### Suggestions

1) Before the intervention of any exercise prescription, due to paying attention to the physical and medication conditions of people with intellectual disabilities, a special person should be assigned to monitor and adjust the exercise content during the exercise. At the same time, the participants' exercise participation motivation, peer demonstration, voice/visual prompt strategy, etc., should be considered. After the exercise, relevant events in the process will be recorded.
2) FITT-VP element of exercise prescription is an important framework for the implementation of exercise training and planning. In addition to reducing obstacles, on-site coaches or fitness instructors can make it easier for people with intellectual disabilities to participate in it and achieve the purpose of improving or enhancing physical fitness.
3) Exercise prescription is not a standardized example. Although the American Academy of Medicine and Sports (ACSM) has developed 51 methods to improve exercise efficiency for patients with chronic diseases or disorders, the common problems of obesity, cardiovascular diseases, metabolic disorders, or medication habits of people with intellectual disabilities make it more complex to formulate appropriate exercise prescription, and the importance of adjusting measures to local conditions cannot be ignored.

## Author Contributions

ZY and PY are responsible for the main research and writing of the article. JL and CQ are responsible for modifying the article. At the same time, ZY is the manager of this research and also the corresponding writer. All authors contributed to the conception and design of this work and approved the final manuscript.

## Funding

This study was supported by the scientific research project of Chongqing Sports Bureau (grant no.: C202013).

## Conflict of Interest

The authors declare that the research was conducted in the absence of any commercial or financial relationships that could be construed as a potential conflict of interest.

## Publisher's Note

All claims expressed in this article are solely those of the authors and do not necessarily represent those of their affiliated organizations, or those of the publisher, the editors and the reviewers. Any product that may be evaluated in this article, or claim that may be made by its manufacturer, is not guaranteed or endorsed by the publisher.
